# Predicting protein-protein interactions with DrugScore^PPI^: fully-flexible docking, scoring, and *in silico* alanine-scanning

**DOI:** 10.1186/1758-2946-3-S1-P36

**Published:** 2011-04-19

**Authors:** DM Krüger, JI Garzón, PC Montes, H Gohlke

**Affiliations:** 1 Department of Mathematics and Natural Sciences, Heinrich-Heine-University, 40225 Düsseldorf, Germany; 2 Centro de Investigaciones Biológicas (CSIC), 28040 Madrid, Spain

## 

Protein-protein complexes play key roles in all cellular signal transduction processes. Here, we present a fast and accurate computational approach to predict protein-protein interactions. The approach is based on DrugScore^PPI^, a knowledge-based scoring function for which pair potentials were derived from 851 complex structures and adapted against 309 experimental alanine scanning results. We developed the DrugScore^PPI^ webserver [1], accessible at http://cpclab.uni-duesseldorf.de/dsppi, that is intended for identifying hotspot residues in protein-protein interfaces. For this, it allows performing computational alanine scanning of a protein-protein interface within a few minutes. Our approach has been successfully validated by application to an external test set of 22 alanine mutations in the interface of Ras/RalGDS and outperformed the widely used CC/PBSA, FoldX, and Robetta methods [1].

Next, DrugScore^PPI^ was teamed with FRODOCK [2], a fast FFT-based protein-protein docking tool, in order to predict 3D structures of protein-protein complexes. When applied to datasets of 54 bound-bound (I) and 54 unbound-unbound (II) test cases, convincing results were obtained (docking success rate for complexes with rmsd < 10 Å: I: ~80%; II: ~50%). Thus, we set out to evaluate whether our approach of deformable potential grids [3], previously developed for protein-ligand docking, also provides an accurate and efficient means for representing intermolecular interactions in fully-flexible protein-protein docking. The underlying idea is to adapt a 3D grid of potential field values, pre-calculated from an initial protein conformation by DrugScore^PPI^, to another conformation by moving grid intersection points in space, but keeping the potential field values constant. Protein movements are thereby translated into grid intersection displacements by coupling protein atoms to nearby grid intersection points by means of harmonic springs and modelling the irregular, deformable 3D grid as a homogeneous linear elastic body applying elasticity theory. Thus, new protein conformations can be sampled during a docking run without the need to re-calculate potential field values.
